# COVID-19 vaccine hesitancy in the Saudi Arabian population

**DOI:** 10.25122/jml-2022-0086

**Published:** 2023-01

**Authors:** Abdullah Almojaibel, Khalid Ansari, Yahya Alzahrani, Maher Alquaimi, Faraz Farooqi, Yousef Alqurashi

**Affiliations:** 1 Respiratory Care Department, College of Applied Medical Sciences, Imam Abdulrahman Bin Faisal University, Dammam, Kingdom of Saudi Arabia; 2 Department of Dental Education, College of Dentistry, Imam Abdulrahman Bin Faisal University, Dammam, Kingdom of Saudi Arabia

**Keywords:** vaccine acceptance, COVID-19 vaccine, coronavirus, Saudi Arabia, vaccine hesitancy

## Abstract

This study aimed to measure the level of vaccine hesitancy among the Saudi population using the WHO Vaccine Hesitancy Scale (VHS). A cross-sectional study using a modified vaccine hesitancy scale (VHS) was conducted among Saudi Arabian residents between April 4 and May 24, 2021. The relationship between participants' willingness to uptake COVID-19 vaccines and their demographics, awareness of COVID-19, and health status was evaluated. The chi-square test was employed to compare categorical variables and logistic regression for the associations of demographical characteristics with acceptance of the vaccine. We received a total of 1657 completed responses. 1,126 participants (68%) were vaccinated, of which 19% were vaccinated with one dose only, and 49% were fully vaccinated (with two doses). Safety concerns and worries about side effects were higher among the hesitant group (p<0.001). 96% of the participants from the willing group were not hesitant to have the vaccine, whereas in the same group, 70% thought they had good health and the vaccine was not needed. Logistic regression analysis revealed that participants with chronic diseases had lower odds of being willing to be vaccinated (OR=0.583, p-value 0.04). The study findings suggest key factors associated with COVID-19 vaccine hesitancy in the Saudi population and can help public health authorities plan strategies to minimize vaccine hesitancy and improve awareness about vaccine acceptance.

## INTRODUCTION

Coronaviruses have been responsible for several outbreaks in recent history, including the Severe Acute Respiratory Syndrome (SARS) outbreak in 2002 and 2003, the Middle East Respiratory Syndrome (MERS) outbreak in 2012, and most recently, the outbreak in 2019 (COVID-19), which was first identified in China, quickly spread worldwide, leading the World Health Organization (WHO) to classify it as a pandemic [1]. As of March 24, 2022, COVID-19 has infected over 472 million people and caused over 6 million deaths globally [2].

The vaccine appeared to be the most effective protection against this virus. However, vaccine hesitancy has been a significant challenge in achieving herd immunity. Several studies have been conducted to investigate factors that influence acceptance rates of COVID-19 among the general population [3–7]. These studies found acceptance rates ranging from 65% to 91%, although most were conducted before vaccination campaigns began and may not reflect current acceptance rates. Higher education, trust in doctors and/or government, older age, working in the governmental sector, being a male, prior influenza infection, and Asian race are some factors associated with a higher acceptance rate.

As of May 24, 2021, the Saudi Ministry of Health (MOH) has administered 13,104,656 COVID-19 vaccine shots to the public in multiple cities and centers. According to the Saudi COVID-19 dashboard, approximately 52% of adults were fully vaccinated with two doses [8]. A high vaccination rate among the public is critical in controlling the spread of the disease and ensuring a sufficient workforce in various sectors. Therefore, the Saudi government planned to vaccinate high-risk individuals in phase 1, with the goal of achieving a 70% vaccination rate in phase 2 [9].

This study aimed to measure the level of vaccine hesitancy among the Saudi population using the WHO Vaccine Hesitancy Scale (VHS). The secondary aim was to assess the attitude toward COVID-19 and the COVID-19 vaccines among the Saudi population.

## MATERIAL AND METHODS

### Design

This cross-sectional study, conducted via an anonymous online self-administered survey, aimed to investigate residents of Saudi Arabia's perceptions of COVID-19 and vaccine hesitancy.

### Sampling

A chain-referral sampling technique was adopted for recruiting the study participants. Survey distribution took place online via links posted on various social media sites such as Twitter and WhatsApp. Participants were encouraged to further distribute the survey among their family, friends, and coworkers. Data collection started on 4/4/2021 and continued until 24/5/2021.

Previous studies on vaccine hesitancy in Saudi Arabia reported that 15% of the studied population was hesitant to take a vaccine [6]. Thus, a sample size of 800 participants is estimated to yield 80% power at a confidence level of 95%. The sample size was calculated using the formula N=Zα^2^ P (1−P)/d^2^, where α=0.05 and Zα=1.96, and the margin of error (d) is 0.025.

### Participants

Regardless of demographic characteristics or history of COVID-19 infection, all adults (>18 years of age) living in Saudi Arabia were eligible for the study. Informed consent was obtained electronically prior to enrollment. Only those who consented to participate were allowed to proceed to the survey. Incomplete responses were excluded from the analysis.

### Measures

The questionnaire included two parts. The first part gathered information about the participant's age, gender, nationality, region of residence, education level, working status, perceived health status, and chronic diseases. Additionally, it explored the participants' views on the severity of the COVID-19 pandemic, worrying level of being infected, their history of COVID-19-related events such as taking PCR tests, and whether they were infected or lived with a family member who was infected with COVID-19.

The second part of the survey assessed the participants' hesitancy to uptake the COVID-19 vaccine measured by a modified version of the vaccine hesitancy scale (VHS) initially developed by the World Health Organization's Strategic Advisory Group of Experts [10]. This modified VHS consists of 10 statements rated by the participants using a 5-point Likert scale (ranging from strongly disagree to strongly agree). The validity and reliability of the modified COVID-19 VHS were previously established [11]. The scale was translated to Arabic using Brislin's model for the back-translation method [12]. The Arabic version was pilot tested on six adults to evaluate the clarity of the items and to establish the scale face validity on the targeted population. For the analysis purpose, a transformation of the VHS scale was done. While comparing the responses, willingness to be vaccinated (item # 10) in the VHS scale was kept as the dependent variable, whereas all other 9 items served as independent variables.

### Statistical analysis

The relationship between participants' demographics, awareness, health status, and willingness to uptake COVID-19 vaccines was evaluated using univariate analyses (frequencies and percentages) and presented in tables. The bivariate analysis included Chi-Square testing for the association of vaccine acceptance with other demographical variables. To identify the potential determinants of the willingness/acceptance (dependent variable with yes or no response) to uptake the COVID-19 vaccines, a multivariate logistic regression model including demographics, health status, and perceptions of COVID-19 infection and vaccines as independent variables was employed. Initially, variables were tested individually for their association with vaccine acceptance, and in the second phase, only those variables that showed significant association were included in the multivariate model.

Furthermore, the survey explored the most common source of information that the participants usually seek for COVID-19 news and updates to establish whether they use reliable sources such as scientific research journals or less reliable ones such as social media or family and friends. Analyses were conducted using SPSS 26.0 (IBM Corporation, New York, NY, United States). The statistical significance level was set at p<0.05.

## RESULTS

**Figure 1:**
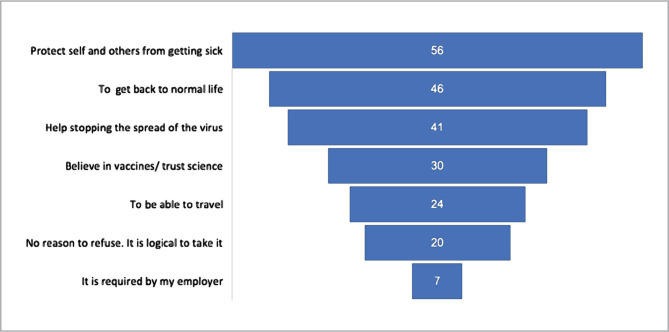
The most common reasons for accepting COVID-19 vaccine.

We received a total of 1871 responses. Of these, 1657 (89%) were fully completed and therefore were analyzed. Table 1 shows the demographical characteristics of the participants. Male participation was higher (58%). Most of the participants (41%) belong to the 18- to 30-year-old age category, live in the Eastern region (76%), have a bachelor's as the highest degree (45%), and are students (30%). Most participants (80%) had no chronic disease, and their perceived health status was perfect (93%). Accepting the vaccine to "protect self and others from getting sick" was the highest-rated reason (56%), followed by wanting "to get back to normal life" (46%) (Figure 1).

**Table 1:** Sociodemographic characteristics of the participants (n=1,657).

Demographic variables		Number (%)
**Gender**	Male	962 (58)
	Female	696 (42)
**Age group**	18–30 years	759 (41)
	31–40 years	387 (21)
	41–50 years	332 (18)
	More than 50 years	393 (21)
**Residency province in Saudi Arabia**	Central Region	225 (14)
	Western Region	123 (7)
	Eastern Region	1,262 (76)
	Southern Region	30 (2)
	Northern Region	17 (1)
**Nationality**	Saudi	1,602 (96.7)
	Non-Saudi	55 (3.3)
**Education level**	Intermediate school or less	60 (4)
	High school	447 (27)
	After high school diploma	177 (11)
	Bachelor's degree	751 (45)
	Higher studies (Masters or Ph.D.)	222 (13)
**Working status**	Health care worker	74 (4)
	Government employee	430 (26)
	Military sector	48 (3)
	Private sector	251 (15)
	Student	491 (30)
	Housewife	87 (5)
	Retired	107 (6)
	I don't work	146 (9)
	Other	23 (1)
**Perception of health status**	Excellent	921 (56)
	Very good	608 (37)
	Intermediate	121 (7)
	Bad	5 (0.3)
	Very bad	2 (0.1)
**Having chronic disease**	Yes	323 (20)
	No	1,333 (80)

At the time of this research, 1,126 (68%) of the participants were vaccinated. Of these, 19% received one dose only, and 49% were fully vaccinated with two doses. Only 15% of the vaccinated group got infected compared to 27% of the non-vaccinated. On the other hand, 78% of the vaccinated group were protected (did not get infected) from COVID-19 infection compared to 61% of the non-vaccinated group. Fewer participants who were not vaccinated (37.2%) than vaccinated (49.9%) believed that COVID-19 is a serious and severe infection (p=0.001). Most (57.6%) vaccinated participants were worried about the infection compared to non-vaccinated (48.2%, p=0.004). 19% of the participants who got the vaccination believed that the virus would be eliminated immediately, whereas 73.9% thought the COVID-19 vaccine would only give some relief from the pandemic (Table 2).

**Table 2:** The association between COVID-19 vaccination status and the participants' beliefs regarding the COVID-19 infection.

Variables		Vaccinated		P-value
		Yes (n=1,126)	No (n=531)	
**Did the polymerase chain reaction (PCR) test**	Yes	67.8%	57.2%	0.001*
	No	32.2%	42.8%	
**Infected with COVID-19**	Yes	14.9%	27.0%	0.001*
	No	78.2%	60.8%	
	Don't know	6.9%	12.2%	
**Family infected with COVID-19**	Yes	83.3%	82.6%	0.773
	No	16.7%	17.4%	
**Beliefs about COVID-19 infection**	A very serious and severe infection	49.9%	37.2%	0.001*
	An infection of some severity	26.8%	30.8%	
	It is similar to common sessional diseases	16.5%	24.6%	
	I have no idea	6.8%	7.4%	
**Worried about being infected**	Very worried	17.6%	13.8%	0.004*
	Intermediately worried	40.0%	34.4%	
	A little worried	23.6%	27.0%	
	Not worried at all	18.8%	24.8%	
**Opinions about the COVID-19 vaccine**	An immediate effect, the virus will be eliminated immediately	18.9%	8.2%	0.002*
	Relief of the pandemic, but the virus will not be eliminated entirely	73.9%	68.0%	
	No effect	2.2%	13.2%	
	I don't know	5.0%	10.6%	

* – Significant (Chi-square test).

A scoring system using the VHS scale was utilized to assess the degree of hesitancy among the Saudi population. The hesitancy scale outcomes were converted from a 5-level scale to a binary system. Responses 1, 2, and 3 from the Likert scale were considered hesitant, while 4 and 5 were not hesitant. Responses regarding willingness to be vaccinated (item 10) were set as the dependent variable and analyzed against all other 9 items (as independent variables) of the VHS. See Table 3 for the logistic regression analysis results. The belief that the COVID-19 vaccine is important for health was significantly higher among the non-hesitant group (65%, p<0.001). However, believing that good health status decreases the need to be vaccinated against COVID-19 was significantly higher among people who are hesitant to be vaccinated. Doubting the safety of the COVID-19 vaccines and being worried about the possible side effects were significantly higher among the hesitant group (p<0.001).

**Table 3:** Willingness to be vaccinated using the VHS items.

VHS Items	Response	I am willing to be vaccinated		P-value
		Not hesitant	Hesitant	
**The COVID-19 vaccine is important for my health**	Not hesitant	96.2%	35.0%	0.0001*
	Hesitant	3.8%	65.0%	
**I am in a good health; I do not need to be vaccinated against COVID-19**	Not hesitant	29.3%	78.6%	0.0001*
	Hesitant	70.7%	21.4%	
**The COVID-19 pandemic has been alleviated, and there is no need to be vaccinated against COVID-19**	Not hesitant	17.1%	71.8%	0.0001*
	Hesitant	82.9%	28.2%	
**I think COVID-19 vaccines will be very effective in preventing COVID-19**	Not hesitant	93.6%	40.8%	0.0001*
	Hesitant	6.4%	59.2%	
**COVID-19 vaccines can protect people (family, friends, colleagues) around me from infection**	Not hesitant	95.3%	43.7%	0.0001*
	Hesitant	4.7%	56.3%	
**I doubt the safety of COVID-19 vaccines**	Not hesitant	40.8%	83.5%	0.0001*
	Hesitant	59.2%	16.5%	
**I am worried about the possible side effects of COVID-19 vaccines**	Not hesitant	65.1%	83.5%	0.0001*
	Hesitant	34.9%	16.5%	
**If the COVID-19 vaccine is recommended by the government, I believe vaccination is beneficial**	Not hesitant	97.7%	44.7%	0.0001*
	Hesitant	2.3%	55.3%	
**The recommendation for the COVID-19 vaccine by doctors, the community, and other professionals has a great influence on me**	Not hesitant	92.9%	36.9%	0.0001*
	Hesitant	7.1%	63.1%	

* – Significant (Chi-square test).

Multivariate logistic regression was performed to explore the demographical factors associated with the hesitancy of getting the COVID-19 vaccine as measured by the question, "would you like to receive it?". The scoring was employed, and responses like "Yes, I would like to, and Yes, but not now" were marked as yes. The other responses, such as "No, I refuse, and I am hesitant", were marked as no. Table 4 shows the logistics regression analysis of the COVID-19 vaccine acceptance model. Age, gender, education level, chronic diseases, family infected, beliefs, worries, and opinions regarding the impact of the COVID-19 vaccine were set as the independent variables. There was no significant difference between males and females regarding the willingness to take the vaccine (p=0.532). However, participants with chronic disease were less likely to get vaccinated than others (OR=0.583, p=0.04). Participants with opinions that the vaccine will have an immediate effect and relieve the pandemic were significantly more likely to get vaccinated (OR=5.94, p=001; OR=2.26, p=0.01, respectively).

**Table 4:** Results of the logistics regression analysis of the COVID-19 vaccine acceptance model.

Variables	OR	95% CL.		P-value
		Lower	Upper	
**Age**				
18–30 years	Ref			
31–40 years	1.147	0.661	1.989	0.626
41–50 years	0.976	0.548	1.738	0.934
More than 50 years	0.73	0.334	1.595	0.43
**Gender**				
Female	Ref			
Male	0.875	0.575	1.33	0.532
**Education**				
Intermediate school or less	Ref			
High school	0.837	0.304	2.303	0.731
After high school diploma	0.441	0.138	1.414	0.168
Bachelor's degree	0.627	0.237	1.656	0.346
Higher studies (Masters or PhD) or equivalent	0.686	0.234	2.013	0.493
**Chronic disease**				
No	Ref			
Yes	0.583	0.349	0.976	0.04*
**Family infected**				
No	Ref			
Yes	0.854	0.497	1.468	0.567
**Believe about COVID-19**				
I have no idea	Ref			
A very serious and severe infection.	1.041	0.445	2.435	0.926
An infection of some severity	0.697	0.3	1.623	0.403
It is similar to common sessional diseases	0.827	0.343	1.995	0.673
**Worried about being infected with COVID-19**				
Not worried at all	Ref			
Very worried	1.478	0.7	3.123	0.306
Intermediately worried	1.397	0.795	2.454	0.245
A little worried	1.491	0.834	2.666	0.178
**Opinions about the impact of the vaccine**				
I don't not know	Ref			
An immediate effect; the virus will be eliminated immediately	5.937	2.128	16.563	0.001*
Relief of the pandemic, but the virus will not be eliminated entirely	2.261	1.184	4.317	0.013*
No effect	0.081	0.025	0.264	0.001*

* – Significant (Wald test).

Figure 1 illustrates the most common reasons cited by participants who were willing to receive the COVID-19 vaccine. Accepting the vaccine to "protect self and others from getting sick" was the highest-rated reason (56%), followed by wanting "to get back to normal life" (46%). The least rated reason to accept the COVID-19 vaccine was that "it was required by the employer". Respondents could choose more than one option from the response categories.

**Figure 2:**
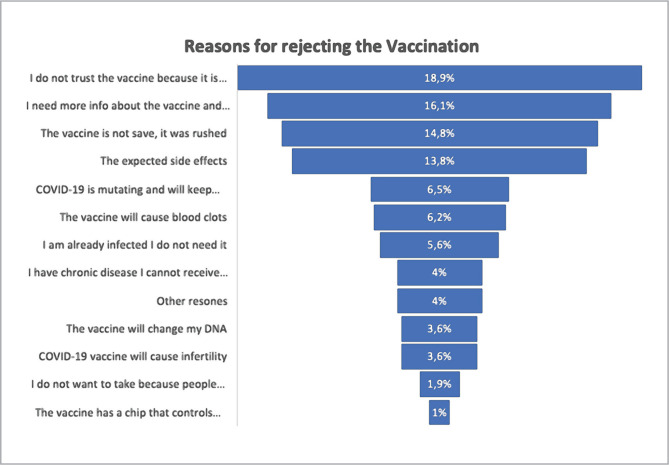
The most common reasons for refusing COVID-19 vaccine.

A total of 213 participants provided reasons for refusing the COVID-19 vaccination. Among them, 19% did not trust the vaccine, 16% still needed more information about the effectiveness of the vaccine, and 15% thought the vaccine was not safe ("it was just implemented without testing"). However, 1% of the participants thought the vaccine had a chip that would control human beings. See Figure 2 for the other reasons for rejection.

**Figure 3:**
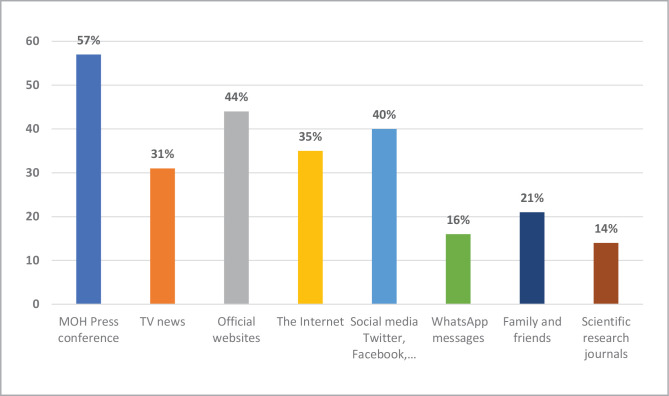
The most common sources of information regarding COVID-19.

Figure 3 represents the most common sources of information about COVID-19 for the Saudi population. Ministry of Health (MOH) press conferences were the most common sources of information about COVID-19 (57%), followed by official websites (44%) and social media (40%). Respondents could choose more than one option from the response categories.

## DISCUSSION

The main finding of this study was that the willingness rate for the COVID-19 vaccine in Saudi Arabia was 68% which is close to the findings from previous studies [13, 14]. However, Ehde *et al*. (2021) [13] included only patients with multiple sclerosis and had a relatively smaller sample size (n=486). Our results are also consistent with Kuter and colleagues (2021), who found a willingness rate of 63.7% among a large sample of hospital employees (n=12034) [14]. In contrast, Guidry *et al*. (2021) reported a much lower willingness rate of 31%. The discrepancy in the results may be due to differences in the age distribution of the samples. Specifically, 41% of the participants in our study were between 18 to 30 years old, while the mean age in Guidry's study was 45.9 years [15].

The positive association between COVID-19 infection and vaccination demonstrates the importance of educating the public about the severity of the infection and the vaccine's efficacy in reducing this severity. These findings are supported by the hypothetic frame explained by Wang *et al*. (2021), who suggested a positive relationship between motivation and perceived severity, perceived efficacy of the COVID-19 vaccine, and confidence in an individual's ability to obtain a vaccine [16]. The above findings highlight the importance of the health ministry and public health authorities in educating the public about the nature of COVID-19 infection and the role of vaccines in limiting its spread. It is crucial to increase the vaccine willingness rate to reach herd immunity and effectively control the pandemic [17–19].

Vaccine hesitancy was higher among those who had doubts about the vaccine safety and the potential side effects of COVID-19 vaccines than among those who did not have any doubt and had minimal concerns about it (83.5% *vs*. 16.5%). These findings are consistent with a study by Syan *et al*. (2021), which reported that 82.8% of the sample were willing to receive a COVID-19 vaccine [20]. In our sample, 78.6% of the Saudi population who believed they were in good health were more hesitant to have the vaccine. The high rate of COVID-19 vaccine hesitancy in the population may be due to a perceived lower risk of infection among individuals without comorbidities, as supported by several studies [21–24]. Additionally, a belief that the pandemic has been mitigated and vaccination is no longer necessary may also contribute to vaccine hesitancy, as seen in the majority (71.8%) of the studied population.

The present study also examined the factors that could predict vaccine hesitancy among the Saudi population. Our data showed that having a chronic illness was a significant predictor for COVID-19 vaccine hesitancy (OR=0.583, p=0.04). This finding is consistent with a study by Bono *et al*. (2021) across nine low- and middle-income countries. In this study, the presence of underlying chronic disease predicted lower odds for willingness to be vaccinated (OR=0.81, p=0.001) [25]. However, these findings contrast other studies which reported that chronic disease was associated with higher rates of COVID-19 vaccine acceptance [3, 26, 27]. Hesitancy to receive the COVID-19 vaccine among participants with chronic diseases could be explained by the perceived side effects of vaccines and negative media messages during early vaccination campaigns.

Furthermore, chronic disease was self-reported and could therefore introduce bias. However, participants who believed that the vaccine would have an immediate effect in reducing the pandemic were more likely to accept the vaccine than those who did not know (OR=5.94, p=0.001). This finding is consistent with previous studies, which have also identified the perceived effectiveness of COVID-19 vaccines as a key factor in vaccine acceptance [28]. Barry *et al*. (2021) also found that healthcare practitioners agreed that the vaccine would stop the pandemic [29].

"This study aimed to examine the factors that influence acceptance of the COVID-19 vaccine among the Saudi population. In line with our results, Yahia *et al*. (2021) also found that the desire to protect oneself and others was the highest-rated reason for accepting COVID-19 vaccines in the Saudi population. Both studies were conducted during a similar timeframe of the pandemic [30]. Our study also investigated reasons for not accepting the vaccine, the most prevalent being a lack of trust in the new vaccine (19%). This is consistent with other studies conducted early in the pandemic, such as one in Hong Kong, which reported high levels of safety concerns (78%) [31], and another in China, where 50% of the population expressed worries about vaccine safety [32]. The fear of adverse side effects in our sample was in line with the rates found in previous studies (11 to 30%) from Saudi Arabia [30, 33, 34].

According to the participants in our study, the most common source of information about COVID-19 vaccines was the Ministry of Health (MOH) press conferences (57%), followed by official websites (44%). The high level of trust in the official sources of information about COVID-19 may explain the vaccine acceptance level among the participants and the low level of mistrust and misinformation among the Saudi population. Other studies also reported high frequencies of using TV and official sources of information to learn about COVID-19 [35, 36]. However, Gecer *et al*. (2020) found that internet journalism was the most commonly used source of information, followed by social media [37]. As suggested by Puri *et al*. (2020), older adults, those with cognitive impairment, lower literacy, and less digital literacy, may be particularly vulnerable to the narrative about vaccines in the media [38]. This highlights the importance of official media channels in Saudi Arabia to develop and disseminate effective vaccine-acceptance messages that target the entire population. Official media channels must also improve their social media presence to promote evidence-based information about COVID-19 infection and vaccines to reach out to the population, including young adults.

The most important limitation of this study was the sample size, which was objectively determined at 800 subjects. However, we used predominantly social media (Twitter, WhatsApp, emails) to outreach study participants during the predetermined collection period. There were some challenges while collecting the data. These include excluding non-social media users, particularly senior citizens [39]. However, only 21% of our sample were over 50 years of age which is relatively less than other studies [40, 41]. Another established limitation of the online survey is biased or inaccurate responses [42]. Nevertheless, the questionnaire used in the study was validated and widely used, and previous studies confirmed that the chance of inaccuracy is minimal, especially with this sample size (n=1693).

Considering these limitations, this study revealed several factors associated with the acceptance or refusal of the COVID-19 vaccine that were previously unrecognized. Additionally, with a sample size that is considered relatively acceptable, our findings indicate a high level of intent among the public to receive the COVID-19 vaccine. Further research is needed to confirm these findings. To promote and increase public vaccination, it is also crucial to investigate public perception of the need and effectiveness of vaccine awareness and promotion programs.

## CONCLUSION

The study's findings highlight key factors associated with COVID-19 vaccine hesitancy among the Saudi population. Participants concerned about the vaccine's safety and potential side effects were more likely to be hesitant about receiving the vaccine. These results can assist public health officials in developing strategies to decrease vaccine hesitancy in Saudi Arabia and create targeted and structured plans to enhance awareness and acceptance of vaccines during this and any future pandemic.

## Data Availability

Further data is available from the corresponding author on reasonable request.
